# The Feasibility of Xpert MTB/RIF Testing to Detect Rifampicin Resistance among Childhood Tuberculosis for Prevalence Surveys in Northern China

**DOI:** 10.1155/2017/5857369

**Published:** 2017-12-05

**Authors:** Jie Lu, Huimin Li, Fang Dong, Jin Shi, Hui Yang, Shujing Han, Ping Chu, Yanlin Zhao, Wenqi Song, Yongli Guo, Shunying Zhao

**Affiliations:** ^1^Beijing Key Laboratory for Pediatric Diseases of Otolaryngology, Head and Neck Surgery, Beijing Pediatric Research Institute, Beijing Children's Hospital, Capital Medical University, National Center for Children's Health, Beijing, China; ^2^Key Laboratory of Major Diseases in Children, Ministry of Education, Beijing Children's Hospital, Capital Medical University, National Center for Children's Health, Beijing, China; ^3^Department 2 of Respiratory Medicine, Beijing Children's Hospital, Capital Medical University, National Center for Children's Health, Beijing, China; ^4^Department of Laboratory Medicine, Beijing Children's Hospital, Capital Medical University, National Center for Children's Health, Beijing, China; ^5^National Center for Tuberculosis Control and Prevention, Chinese Center for Disease Control and Prevention, Beijing, China

## Abstract

Drug resistance surveillance is crucial for control of drug-resistant tuberculosis (TB). However, limited data exists on the burden of drug-resistant TB in children. The goal of this work was to generate prevalence data regarding rifampicin- (RIF-) resistant childhood TB in northern China and to test the feasibility of Xpert for surveying pediatric TB drug resistance prevalence. We enrolled 362 clinically diagnosed childhood TB patients and collected sputum, gastric lavage aspirate (GLA), bronchoalveolar lavage fluid (BALF), and cerebral spinal fluid (CSF) samples. Xpert and solid culture were utilized to detect RIF resistance. The detection rate of Xpert-positive TB among new clinically diagnosed TB cases was 38.4% (139/362), significantly higher than that of solid culture-positive TB (16.3%, 59/362, *P* < 0.01). Notably, Xpert-positive rates differed significantly by sample type, with the highest positive rate for GLA (51.2%). The unit testing costs per RIF-resistant TB patient were $828.41 for solid culture and $761.86 for Xpert. Our data demonstrate that the prevalence of RIF resistance among childhood TB cases in our study (6.9%) is comparable to the national RIF resistance prevalence level of new cases (6.7%). In addition, Xpert is superior to the solid culture for RIF resistance survey in the childhood TB patients.

## 1. Introduction

The threat of childhood tuberculosis (TB) is increasingly being recognized as a major public health concern [1, 2]. The World Health Organization (WHO) estimates that in 2015 one million new pediatric tuberculosis cases were diagnosed, accounting for approximately 10% of the global TB burden [3]. In settings where TB is endemic, such as India and China, the estimated number of children with this disease constitutes 20% of all active TB cases [2]. Because children with TB will transmit disease to future generations [2], more attention should be devoted to minimizing childhood TB in order to reduce the future overall global TB burden.

Surveillance of drug-resistant TB is crucial to formulate effective interventions to prevent emergence of drug resistance and to effectively prevent TB transmission to the community [4]. Unfortunately, due to the paucibacillary nature of pediatric TB, children are not usually included in regional and national surveys of drug-resistant TB incidence and prevalence [5, 6]. As a consequence, only limited data on the burden of drug-resistant TB in children is available globally [5], resulting in failures of not only early diagnosis and effective treatment, but also long-term management of drug-resistant TB in this special population. Therefore, to minimize the contribution of childhood TB to future TB disease burden, management of TB in children should urgently focus on control of drug-resistant TB.

China is undoubtedly the global “hotspot” of drug resistance [3, 7]. A national drug resistance survey revealed that 5.7% of new adult cases and 25.6% of previously treated adult cases harbored multidrug-resistant TB (MDR-TB) [7]. However, TB patients under 15 years of age were excluded from this survey due to the absence of pragmatic strategies offered by the National Tuberculosis Programme (NTP) of China to guide the management of pediatric TB cases [8]. In China, pediatric TB patients are distinct from their adult counterparts in that diagnosis and treatment of pediatric TB patients are mainly provided by children's hospitals rather than specialized TB hospitals. Because laboratories within children's hospitals usually cannot comply with biosafety requirements for conventional drug susceptibility testing (DST), the lack of antibiotic resistance data for MTB* (Mycobacterium tuberculosis)* isolates from their patients has greatly hindered implementation of drug resistance surveillance. Recently, WHO has recommended the use of the Xpert MTB/RIF assay for surveillance purposes in resource-limited areas [9, 10]. Because northern China may be viewed as a resource-limited area, the Xpert assay was adopted herein to study pediatric TB patients in this region. The goals of this study were (1) to obtain data on prevalence of RIF-resistant childhood TB and (2) to test the feasibility of using Xpert to collect data for drug resistance prevalence surveys of childhood TB.

## 2. Methods

### 2.1. Ethics Statement

This study was approved by the Ethics Committee of Beijing Children's Hospital, Capital Medical University (Ethics Committee Approval Number 2013-025). The methods used in this study were performed in accordance with relevant guidelines and regulations, approved by our institute and national government. For all of the 831 patients enrolled in this study, written informed consent was obtained from their guardians on their behalf.

### 2.2. Patient Enrollment

We performed a prospective study between January 2013 and December 2015 of pediatric inpatients under 15 years of age at Beijing Children's Hospital with symptoms of presumptive TB. All inpatients admitted to internal medicine wards were screened for presumptive TB if they exhibited at least one of the following symptoms: cough for more than 2 weeks, fever for more than 2 weeks, TB contact history, and radiographic features suggestive of pulmonary TB. Pediatric physicians who had majored in respiratory medicine conducted the screening and determined which patients had presumptive TB requiring additional follow-up. Demographic information was obtained by interviewing the children's guardians and HIV testing was routinely performed. The HIV-positive patients and patients without complete clinical data or valid samples suitable for further analysis were excluded from the study.

All of the enrolled patients were examined by chest X-ray (CXR), tuberculin skin test (TST), acid-fast-bacilli (AFB) smear microscopy, and solid culture. CXR features consistent with childhood TB included mediastinal and/or hilar lymphadenopathy with pulmonary parenchymal infiltration, bronchial obstruction, cavitary or miliary lesions, and calcification. The Mantoux TST was performed according to standard protocols using five tuberculin units of purified protein derivative (PPD). The results were read between 48 and 72 hours after injection and indurations with a diameter of 5 or more millimeters were considered positive.

TB cases were diagnosed as described in previous studies with minor modifications [8, 11, 12]. Two independent pediatric physicians majored in respiratory medicine made the primary diagnosis and an expert group within Beijing Children's Hospital consisting of senior physicians who were experienced in childhood TB made the final diagnosis. The study population included both pulmonary TB and TB meningitis. “Confirmed TB” cases were defined as children with cough and fever for >2 weeks and CXR features consistent with TB and positive solid culture. “Clinically diagnosed TB” cases were defined as children with cough and fever for >2 weeks and CXR features consistent with TB and who met at least two of the following criteria: (1) contact history with active TB patients, (2) positive TST results, or (3) anti-TB therapy being effective. “Patients with pulmonary TB combined with TB meningitis” were defined as patients with confirmed TB or clinically diagnosed TB combined with clinical signs of meningitis and characteristic CSF findings (macroscopically clear with pleocytosis, elevated protein, and reduced glucose).

### 2.3. Sample Collection

All specimens were collected for diagnostic purposes. Four types of specimens were collected from clinically diagnosed TB patients including sputum (expectorated or induced) from patients with pulmonary TB, gastric lavage aspirate (GLA) or bronchoalveolar lavage fluid (BALF) from sputum-scarce patients, and cerebral spinal fluid (CSF) from patients with pulmonary TB combined with TB meningitis. The clinical samples were simultaneously divided into two duplicate groups for parallel mycobacterial culture and Xpert examination. One group of specimens was tested directly by AFB smear microscopy followed by solid culture and the duplicate group was sent to the Chinese Center of Disease Control and Prevention (Chinese CDC) for Xpert testing.

### 2.4. Laboratory Examination

AFB smear microscopy was performed according to the WHO standard protocol. The remainder of each specimen was decontaminated with N-acetyl-L-cysteine-NaOH (NALC-NaOH) for 15 minutes to remove nonmycobacterial microorganisms. Next, the digested samples were inoculated onto modified solid Löwenstein-Jensen (L-J) media according to previously reported methods [13]. Bacterial colonies were harvested from the L-J media and sent to the Chinese CDC for conventional drug susceptibility testing and species identification [14]. The concentration of RIF in media was 40 *μ*g/mL. According to guidelines recommended by WHO, the strains were declared resistant to RIF when the growth rate was more than 1% of that of the control. In addition, paranitrobenzoic acid (500 mg/mL) and thiophene-2-carboxylic acid hydrazide (5 mg/mL) were used for the purpose of mycobacterium species identification.

For each cultured specimen, a duplicate specimen was subjected to Xpert testing. Briefly, 1 mL of each clinical specimen, with the exception of GLA samples, was mixed with 2 mL Xpert MTB/RIF sample reagent and incubated at room temperature for 15 min. GLA samples, prior to testing by Xpert, were neutralized with an equal volume of sterile 1% sodium bicarbonate. After incubation of all sample types, 2 mL of each treated specimen was added to a test cartridge and loaded onto the Xpert instrument. The bacterial load levels determined for the samples were categorized according to the Xpert reports and included high, medium, low, and very low levels.

### 2.5. Economic Evaluation

Laboratory costs associated with Xpert, solid culture, and DST were obtained from a previous Chinese study and were estimated using the time-observation method [15, 16]. All costs were collected during a time period from a specimen digested at the lab until test results were available, including costs for building, laboratory space, equipment, reagents, consumables, and staff salary, as well as quality control and equipment maintenance. Various methods, such as investigation, interview, and field collection, were utilized to obtain the cost data. According to the literature [16], unit costs (including overhead, building, equipment, staff, reagents, and chemicals) of Xpert, solid culture, and DST were 118.62, 47.87, and 159.71 RMB, respectively. The overall experimental costs of Xpert and conventional methods were calculated according to the numbers of assays used in this study. Because we needed to transport samples for Xpert and bacterial cultures/plates for DST to the Chinese CDC, these transport costs were added to total costs of Xpert and DST. In addition, we analyzed the cost of identifying a single case of RIF-resistant tuberculosis by dividing the total cost by the number of RIF-resistant cases detected using different methods. A currency conversion rate of 670 RMB per 100 US dollars was used in the data analysis.

### 2.6. Statistical Analysis

Bacterial culture methods (liquid culture over solid L-J culture) are considered the gold standard for detection of TB. However, due to limited laboratory resources, we could only perform solid L-J culture in our hospital. Thus, in this study we used two different reference standards: (1) solid culture and (2) clinical diagnosis. The sensitivity of Xpert was calculated using culture-positive TB as the denominator, and TB-positive rates determined using Xpert and solid L-J culture were calculated using number of clinically diagnosed TB cases as the denominator.

Statistical analyses were performed using SPSS 19.0 software (SPSS Inc., Chicago, IL, USA) and SAS 9.3 software (SAS Institute Inc., Cary, NC, USA). Categorical data were presented as the number of cases and percentages. Pearson Chi-square test or Fisher's exact test (where cell counts were low) was performed to assess the effect of various risk factors. Relative risk (RR) and 95% confidence intervals (CIs) were calculated for each of the categorical variables and risk factors with statistical significance were included in further analysis using log-binomial regression analysis. Stepwise multivariate log-binomial analysis was used to examine the association between risk factors and Xpert results, adjusting for confounding risk factors. *P* values less than 0.05 were considered statistically significant.

## 3. Results

### 3.1. Xpert

**Figure 1:**
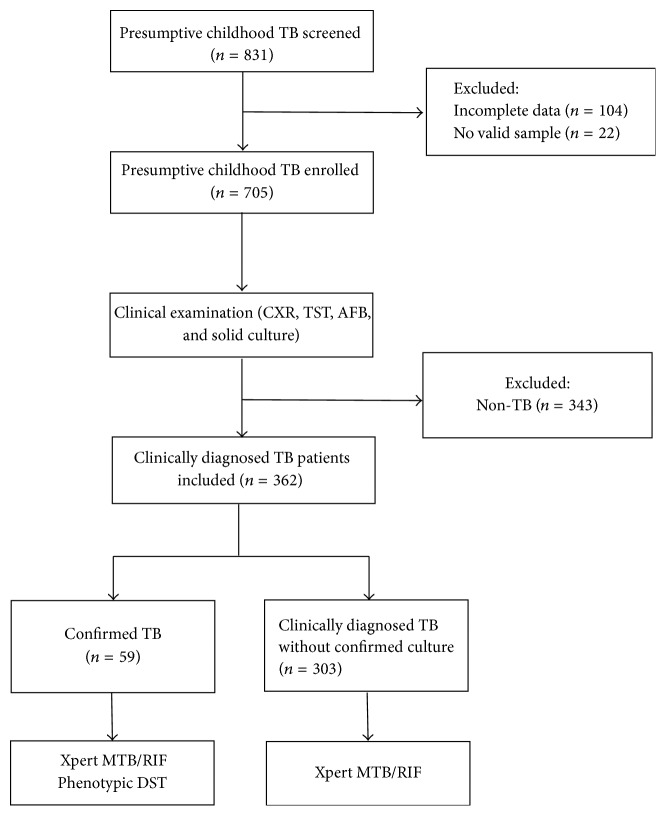
Flow diagram of patient enrollment.

**Figure 2:**
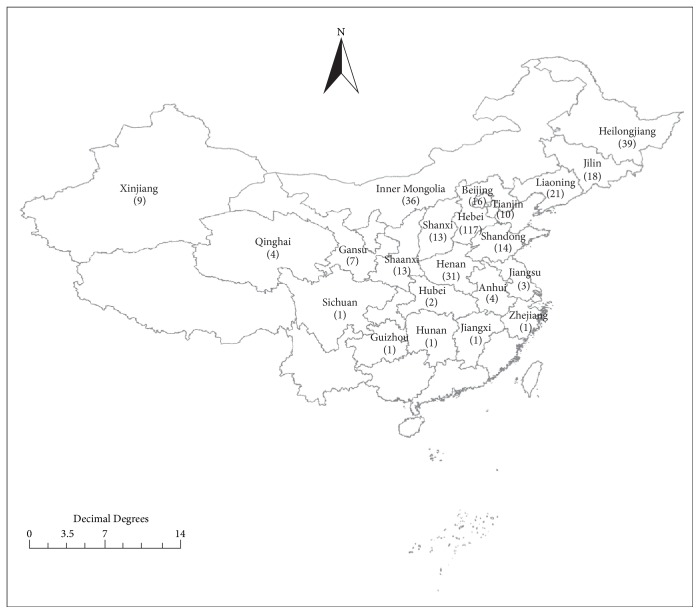
Distribution of childhood TB patients enrolled in this study. The number annotated in the figure represents the number of childhood TB patients from each province. The background map was created using Matlab 7.0 software (The MathWorks, MA, USA, https://www.mathworks.com/products/matlab), Adobe Photoshop 6.0 (Adobe Systems Inc., CA, USA, http://www.adobe.com/cn/products/photoshop.html), and Adobe Illustrator CS4 (Adobe Systems Inc., CA, USA, http://www.adobe.com/products/illustrator.html).

A total of clinically diagnosed 362 childhood TB patients (ages ranging from 1 month to 14 years) were enrolled in this study, including 59 (16.3%) confirmed TB cases with positive results of solid culture and 303 (83.7%) clinically diagnosed TB cases without confirmed culture (Figure 1). Patients included 219 males and 143 females. As shown in Figure 2, the patients originated from 22 provinces in northern China. Among these provinces, Hebei Province contributed the largest number of patients, accounting for 32.3% of all patients (117/362). The next two largest patient groups originated from Heilongjiang (39/362, 10.8%) and Inner Mongolia (36/362, 9.9%).

Xpert and solid culture were performed for all the 362 TB patients. Using solid culture as a reference standard, the sensitivity of Xpert for TB detection was 88.1% (52/59). Using clinical diagnosis as a reference standard, the TB-positive rate of Xpert was 38.4% (139/362), which was significantly higher than that of solid L-J culture (16.3%, 59/362, *P* < 0.01). In addition, Xpert quantitation results for the 139 positive specimens were “high” for 3 (2.2%), “medium” for 22 (15.8%), “low” for 44 (31.6%), and “very low” for 70 (50.4%). Furthermore, among the 139 Xpert-positive samples, 87 (62.6%) samples were culture-negative, of which 24 were “low” (27.6%, 24/87) and 63 were “very low” (72.4%, 63/87) using Xpert quantitation.

**Figure 3:**
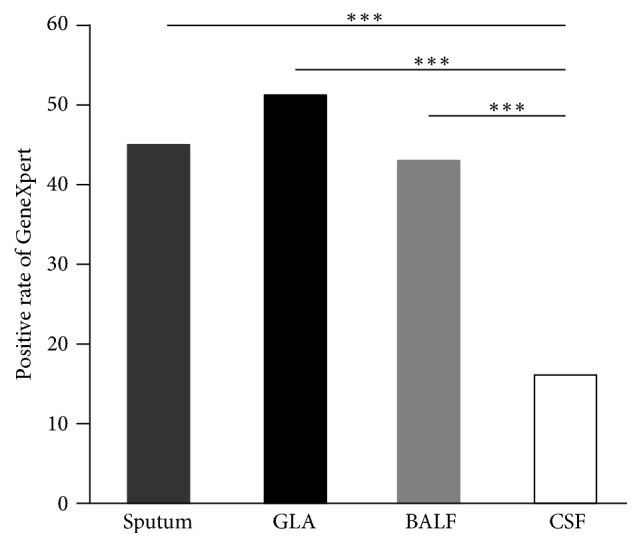
Comparison of the positive rates of Xpert in different samples. GraphPad Prism 5.03 (GraphPad Software Inc., San Diego, CA, USA) was used to generate the figure. *∗∗∗* indicated *P* < 0.001.

We further analyzed the positive rate of Xpert according to the different specimen types for the 362 patients. Sputum, GLA, BALF, and CSF were individually obtained from 47 (13.0%), 127 (35.1%), 86 (23.7%), and 102 (28.2%) patients. As shown in Figure 3, the highest positive rate was observed in GLA samples, with a positive rate of 51.2% (65/127). In addition, out of 47 sputum samples and 86 BALF samples, 21 (44.7%) and 37 (43.0%) were identified as Xpert-positive. In contrast, only 16 (15.7%) positive samples were detected from 102 CSF specimens by Xpert, and the positive rate of CSF was significantly lower than that of GLA (*P* < 0.001), BALF (*P* < 0.001), or sputum (*P* < 0.001). The culture-positive rates in GLA, sputum, BALF, and CSF samples were 19.7% (25/127), 19.1% (9/47), 17.4% (15/86), and 9.8% (10/102), respectively.

### 3.2. Risk Factors Associated with Xpert-Positive TB Cases

Classification of childhood patients was shown in Table 1, which was stratified according to gender, age, residence, and contact history. The univariate analysis using Pearson Chi-square test or Fisher's exact test showed that female gender (RR = 1.55, 95% confidence interval [CI] = 1.01–2.40, and *P* = 0.04), age younger than one year (RR = 2.94, 95% CI = 1.69–5.13, and *P* < 0.01), and residence in rural region (RR = 3.98, 95% CI = 2.27–7.01, and *P* < 0.01) were significant risk factors for Xpert-positive TB. The multivariate analysis using log-binomial revealed that age younger than one year (RR = 1.24, 95% CI = 1.15–1.35, and *P* = 0.04) and residence in rural region (RR = 1.27, 95% CI = 1.16–1.39, and *P* < 0.01) were significantly associated with Xpert-positive results. In both the univariate and multivariate analyses, age younger than one year and residence in rural region significantly increased the risk for an Xpert-positive result. In addition, we found that the contact history and treatment history were not significantly associated with Xpert-positive results in both the univariate and multivariate analyses (*P* > 0.05) and thus had no influence on the distribution of Xpert-positive cases.

### 3.3. Prevalence of RIF Resistance

Of the 139 Xpert-positive samples, 9 (6.5%) were detected as RIF-resistant and 121 (87.0%) as RIF-susceptible. Meanwhile, the other 9 samples (6.5%) were flagged as “RIF resistance Indeterminate,” which were all from the “very low” bacillary load group, suggesting that the failure to detect RIF resistance may be due to the low bacillary loads in these samples. We also analyzed the proportion of indeterminate RIF resistance samples by sample type. As shown in Table 2, the rate of indeterminate RIF resistance in CSF samples (25.0%, 4/16) was significantly higher than that for GLA (3.1%, 2/65, *P* = 0.01), while there was no significant difference among GLA, BALF, and sputum groups (*P* > 0.05). When the samples with indeterminate RIF resistance were excluded from the final analysis, our data indicated that the prevalence of RIF-resistant tuberculosis among childhood patients in our study was 6.9% (9/130). In addition, when 59 culture-positive samples were analyzed using the conventional proportional DST test, five of 59 were RIF-resistant, which were all detected by the Xpert assay.

### 3.4. Cost of Xpert and Solid Culture Testing for Detecting RIF-Resistant TB

The total transport costs of the 362 samples for Xpert and 59 bacterial colonies for DST were 3,000 and 1,000 RMB, respectively. The total costs for the various methods were obtained after addition of transport costs and unit costs were calculated by dividing the total cost by the number of positive cases.

Using solid culture as reference standard, the unit costs by Xpert for each Xpert-positive TB and RIF-resistant childhood TB were $131.86 and $1371.36, respectively. Using clinical diagnosis as reference standard, the unit costs for each RIF-resistant TB childhood patient were $828.41 for solid culture and $761.86 for Xpert (Table 3). In addition, the unit cost of both solid culture and Xpert differed by sample type. For solid culture, the highest cost per RIF-resistant case was observed in BALF samples ($1009.96), while the unit cost for a sputum sample was the lowest ($573.11). For Xpert, the unit cost of the RIF resistance test for a CSF sample was the highest ($996.01), while the cost for the GLA sample was the lowest ($601.39).

## 4. Discussion

Surveillance for drug resistance is crucial for prevention and control of drug-resistant TB [4]. However, children are not usually included in regional and national surveys of drug-resistant TB incidence and prevalence due to the disease's paucibacillary nature; thus data on the burden of drug-resistant TB in children is limited globally [5, 6]. In this study, we first incorporated the Xpert assay into our overall strategy for drug survey of childhood TB patients. Our data revealed that Xpert is superior to conventional culture methods, achieving a higher positive detection rate of pediatric TB cases from various clinical samples. The total positive rate of Xpert is 38.4% in our study, using clinical diagnosis as reference standard. In contrast to our findings, several previous evaluation studies showed that bacterial culture methods had higher positive rates for smear positive and smear negative TB cases [17, 18]. There are several potential reasons that could account for this difference. On one hand, the conventional L-J culture, rather than liquid culture, was used in the present study due to limited resources in our hospital. Consequently, the relatively low performance of mycobacterial recovery using solid culture may underestimate the number of bacterium-positive TB patients. On the other hand, the high proportion of GLA samples in this study may be another important cause of low detection rate when using solid culture. Because of children's difficulties in expectorating sputum, most of the samples obtained from childhood TB patients are GLA samples. In spite of their demonstrated tolerance of acidic environments, most mycobacteria are killed after a long period of exposure to gastric acid, thereby resulting in low sensitivity observed for pediatric GLA samples. In agreement with our hypothesis, a recent study from Pang et al. found that the detection rate of conventional culture from GLA samples was lower than the rate obtained using the Xpert assay [8]. Hence, our findings highlight that Xpert serves as an alternative for the diagnosis of childhood TB with GLA samples. Notably, positive rates of detection of TB using the Xpert assay differed significantly by sample type. Compared with other samples, CSF showed the lowest detection rate for Xpert-positive cases, which seemed to be linked to the low bacillary load in CSF samples [19]. In addition, the low positive rate in CSF samples yielded a high average cost for detection of RIF-resistant cases using Xpert, which was 60% higher than the average cost for GLA samples. Thus, the Xpert assay might not be a suitable tool for drug resistance survey using CSF samples.

The bacillary load is always used as a marker of disease severity [20]; thus Xpert-positive patients may be more serious when compared with Xpert-negative patients. Our results have shown that the patients from rural region were more likely to have a positive Xpert result, which may be associated with the delay of seeking healthcare due to unsatisfactory health resource settings in the rural regions [21]. Compared with male children, female TB children had higher proportion of GeneXpert positive cases. In China, because of traditional value system, girls receive less attention than boys and as a result suffer from higher mortality from diseases [22]. Hence, we hypothesize that the female TB children in China also have to face this dilemma regarding the diagnosis delay, leading to the higher rate of GeneXpert positive in this population. In addition, we found that infants aged less than 1 year were also a risk factor for yielding positive Xpert results. In view of limited exposure to antigens in utero, infants are thought to have compromised immunity for protection against infections [23]. Notably, the poor immunity in infants is associated with the progression of tuberculosis, which may be responsible for the higher frequency of bacterium-positive cases in infants. Similarly, Yin and colleagues showed that age was significantly associated with the positive rate of Xpert assay and the Xpert-positive rate of patients younger than 3 years of age was significantly higher than that of cases older than 3 years of age [24].

RIF resistance is considered a surrogate marker for multidrug-resistance and poor treatment outcomes [25]. Here, we found that the prevalence rate of RIF resistance among childhood TB cases in northern China was 6.9%, which was similar to that of pediatric TB cases from Chongqing (5.1%) [26]. However, the prevalence rate in the present study was much lower than that of the study by Jiao et al. using childhood TB strains collected from different regions of China (23.3%) [27]. The differences observed among various reports might be mainly attributed to enrollment differences in the proportion of TB cases with previous treatment histories. As mentioned in the national survey of drug-resistant TB in China [7], the RIF-resistant rate among new cases (6.7%) was much lower than that for previously treated cases (29.4%). The samples in the study by Jiao and colleagues consisted of 73% previously treated cases [27], while in our study more than 95% of patients were new cases, as in the study from Chongqing. Thus, it is reasonable to assume that the prevalence rate of RIF resistance herein was similar to that in Chongqing [26]. Interestingly, the RIF resistance rate of childhood TB in our study is comparable with the prevalence rate of RIF resistance among new cases (6.7%), as determined by the national survey in China. Although the Chinese national survey was done in an adult population, available evidence suggests the prevalence of drug-resistant TB in children is similar to that of adults [28].

In China, due to the absence of childhood TB from National Tuberculosis Programme (NTP), the diagnosis and treatment of childhood TB patients usually rely on the children's hospitals rather than TB dispensaries or TB specialized hospitals. Unfortunately, children's hospitals rarely have the capability to perform mycobacterial culture; thus the diagnosis of childhood TB mainly depends on sputum-smear microscopy in most children's hospitals in China. Moreover, the lack of positive culture results makes it difficult to carry out drug susceptibility testing. In addition, due to the acquirement of higher biosafety level laboratory, most children's hospitals in China could not perform DST based on culture. Therefore, DST testing requires transportation of samples or bacteria colonies from children's hospitals to DST laboratories. On the other hand, Xpert can be performed on-site. Moreover, the intrinsic advantages of Xpert testing make it more suitable for application to drug survey of childhood TB in nonspecialized hospitals, such as children's hospitals. First, in contrast to mycobacterial culture, Xpert employs analysis of nucleic acid sequences, does not require viable bacilli, and is less sensitive to specimen storage and transport conditions [29]. Second, the automated performance and accessibility required in laboratory settings allow Xpert to be incorporated into the current diagnostic algorithm more conveniently and accurately. Third, the higher detection rate of Xpert is associated with more information regarding drug resistance of childhood TB as well as a lower cost for survey of drug resistance. Taken together, the data presented here demonstrate that Xpert is more cost-effective than other methods, allowing drug resistance survey of childhood TB cases to be performed in China.

There were several limitations in this study. First, this study was carried out in only one hospital in China. Although more than 60% of patients seeking healthcare in this hospital originated from all regions in northern China, the sampling of only one hospital no doubt resulted in sampling bias. Second, the diagnosis of childhood TB was very difficult. Thus, we made the clinical diagnosis using the diagnostic standards according to Chinese Medical Association, which were stricter than expert consensus on tuberculosis diagnostics in children published in 2012 [30]. However, we should still admit that a few of the 362 TB patients might not be real TB patients, and some of them might have clinical improvement after anti-TB treatment. Third, due to the small sample size of RIF-resistant cases, the risk factors associated with RIF resistance were not included in the final analysis. Finally, the current Xpert assay can only detect RIF resistance but cannot detect other types of drug resistance. However, the next generation of the Xpert assay will incorporate detection of resistance to isoniazid, fluoroquinolone, and second-line injectable drugs in addition to RIF. Despite these current limitations, our study data show that the Xpert assay is a promising alternative for drug resistance surveillance in resource-limited settings.

In conclusion, our findings demonstrate that Xpert effectiveness exceeds conventional culture methods for performing RIF resistance surveillance in pediatric TB patients. Using Xpert, the prevalence of RIF resistance among new childhood tuberculosis cases in northern China was found to be comparable to the national RIF resistance rate throughout China. It must be noted, however, that the detection rate of the Xpert assay differs significantly by sample type. It should also be emphasized that female patients, patients from rural regions, and patients younger than 1 year of age are at higher risk of positive Xpert results. Additional research using multicenter studies is needed to further assess the feasibility of Xpert for drug resistance surveillance throughout China.

## Figures and Tables

**Table 1:** Demographic characteristic of childhood TB patients enrolled in this study.

Characteristics	Diagnostic class			Univariate		Multivariate log-binomial	
	Xpert-positive (139) *N* (%)	Xpert-negative (223) *N* (%)	Total (362) *N* (%)	Relative risk (95% CI^*∗*^)	*P* value	Relative risk (95% CI^*∗*^)	*P* value
*Gender*							
Male	75 (54.0)	144 (64.6)	219 (60.5)	1.00	—	1.00	—
Female	64 (46.0)	79 (35.4)	143 (39.5)	1.55 (1.01–2.40)	0.04	1.08 (0.99–1.19)	0.11
*Age (years)*							
<1	59 (42.4)	53 (23.8)	112 (30.9)	2.94 (1.69–5.13)	<0.01	1.24 (1.15–1.35)	0.03
1–5	31 (22.3)	82 (36.8)	113 (31.2)	1.00	—	1.00	—
>5	49 (35.3)	88 (39.4)	137 (37.9)	1.47 (0.87–2.53)	0.16	1.15 (0.83–1.43)	0.24
*Residence*							
Urban	18 (12.9)	83 (37.2)	101 (27.9)	1.00	—	1.00	—
Rural	121 (87.1)	140 (62.8)	261 (72.1)	3.98 (2.27–7.01)	<0.01	1.27 (1.16–1.39)	<0.01
*Contact history*							
No	92 (66.2)	144 (64.6)	236 (65.2)	0.93 (0.60–1.45)	0.75	—	—
Yes	47 (33.8)	79 (35.4)	126 (34.8)	1.00	—	—	—
*Treatment history*						—	—
New cases	135 (97.1)	215 (96.4)	350 (96.7)	1.00	—	—	—
Retreated	4 (2.9)	8 (3.6)	12 (3.3)	0.80 (0.24–2.70)	0.77	—	—

^*∗*^CI: confidence interval.

**Table 2:** RIF resistance determined by GeneXpert.

Specimen^a^	RIF resistance determined by GeneXpert (%)			
	Resistant	Susceptible	Indeterminate	Total
Sputum	1 (4.8)	19 (90.4)	1 (4.8)	21 (100.0)
GLA	4 (6.1)	59 (90.8)	2 (3.1)	65 (100.0)
BALF	2 (5.4)	33 (89.2)	2 (5.4)	37 (100.0)
CSF	2 (12.5)	10 (62.5)	4 (25.0)	16 (100.0)
Total	9 (6.5)	121 (87.0)	9 (6.5)	139 (100.0)

^a^GLA, gastric lavage aspirate; BALF, bronchoalveolar lavage fluid; CSF, cerebrospinal fluid.

**Table 3:** Comparison of costs (USD) on detecting RIF-resistant childhood TB patients from different specimen types.

		Specimen types				
		CSF	GLA	BALF	Sputum	Average
Gold standard 1 = solid culture	*Per positive case*					
	Xpert	276.00	100.23	125.30	111.28	131.86
	*Per RIF-R* ^*∗*^ * case*					
	Xpert	1932.02	1202.78	1628.96	890.25	1371.36

Gold standard 2 = clinical diagnosis	*Per positive case*					
	Solid culture	72.88	36.30	40.96	37.31	43.84
	Xpert	120.75	37.01	44.03	42.39	49.33
	*Per RIF-R* ^*∗*^ * case*					
	Solid culture	992.44	783.28	1009.96	573.11	828.41
	Xpert	966.01	601.39	814.48	890.25	761.86

^*∗*^RIF-R: rifampicin-resistant.

## References

[1] Dodd P. J., Sismanidis C., Seddon J. A. (2016). Global burden of drug-resistant tuberculosis in children: a mathematical modelling study. *The Lancet Infectious Diseases*.

[2] Perez-Velez C. M., Marais B. J. (2012). Tuberculosis in children. *The New England Journal of Medicine*.

[3] World Health Organization (2016). *Global Tuberculosis Report 2016*.

[4] Wright A., Zignol M., Van Deun A. (2009). Epidemiology of antituberculosis drug resistance 2002-07: an updated analysis of the Global Project on Anti-Tuberculosis Drug Resistance Surveillance. *The Lancet*.

[5] Williams B., Ramroop S., Shah P. (2013). Multidrug-resistant tuberculosis in UK children: Presentation, management and outcome. *European Respiratory Journal*.

[6] Sandgren A., Cuevas L. E., Dara M. (2012). Childhood tuberculosis: Progress requires an advocacy strategy now. *European Respiratory Journal*.

[7] Zhao Y., Xu S., Wang L. (2012). National survey of drug-resistant tuberculosis in China. *The New England Journal of Medicine*.

[8] Pang Y., Wang Y., Zhao S., Liu J., Zhao Y., Li H. (2014). Evaluation of the Xpert MTB/RIF assay in gastric lavage aspirates for diagnosis of smear-negative childhood pulmonary tuberculosis. *The Pediatric Infectious Disease Journal*.

[9] Tahseen S., Qadeer E., Khanzada F. M. (2016). Use of Xpert® MTB/RIF assay in the first national antituberculosis drug resistance survey in Pakistan. *The International Journal of Tuberculosis and Lung Disease*.

[10] World Health Organization (2015). *Guidelines for Surveillance of Drug Resistance in Tuberculosis*.

[11] Li H., Lu J., Liu J., Zhao Y., Ni X., Zhao S. (2016). Linezolid is associated with improved early outcomes of childhood tuberculous meningitis. *The Pediatric Infectious Disease Journal*.

[12] (2006). S. o. P. Subspecialty Group of Respiratory Diseases, Subspecialty Group, of Respiratory Diseases, Chinese Medical Association. Diagnostic standards and therapeutic recommendations for pulmonary tuberculosis in children. *Chinese journal of pediatrics*.

[13] Pang Y., Xia H., Zhang Z. (2013). Multicenter evaluation of genechip for detection of multidrug-resistant mycobacterium tuberculosis. *Journal of Clinical Microbiology*.

[14] Pang Y., Zhou Y., Zhao B. (2012). Spoligotyping and drug resistance analysis of Mycobacterium Tuberculosis strains from national survey in China. *PLoS ONE*.

[15] Pang Y., Li Q., Ou X. (2013). Cost-Effectiveness Comparison of Genechip and Conventional Drug Susceptibility Test for Detecting Multidrug-Resistant Tuberculosis in China. *PLoS ONE*.

[16] Ou X., Xia H., Li Q. (2013). Cost analysis of Xpert Mtb/RIF test for the detection of M. tuberculosis and rifampin resistance. *Chinese Journal of Antituberculosis*.

[17] Nicol M. P., Workman L., Isaacs W. (2011). Accuracy of the Xpert MTB/RIF test for the diagnosis of pulmonary tuberculosis in children admitted to hospital in Cape Town, South Africa: a descriptive study. *The Lancet Infectious Diseases*.

[18] Boehme C. C., Nabeta P., Hillemann D. (2010). Rapid molecular detection of tuberculosis and rifampin resistance. *The New England Journal of Medicine*.

[19] Wang T., Feng G.-D., Pang Y. (2016). High rate of drug resistance among tuberculous meningitis cases in Shaanxi province, China. *Scientific Reports*.

[20] Bates M., O'Grady J., Maeurer M. (2013). Assessment of the Xpert MTB/RIF assay for diagnosis of tuberculosis with gastric lavage aspirates in children in sub-Saharan Africa: a prospective descriptive study. *The Lancet Infectious Diseases*.

[21] Cheng G., Tolhurst R., Li R. Z., Meng Q. Y., Tang S. (2005). Factors affecting delays in tuberculosis diagnosis in rural China: A case study in four counties in Shandong Province. *Transactions of the Royal Society of Tropical Medicine and Hygiene*.

[22] Banister J. (2004). Shortage of girls in China today. *Journal of Population Research*.

[23] Prabhudas M., Adkins B., Gans H. (2011). Challenges in infant immunity: Implications for responses to infection and vaccines. *Nature Immunology*.

[24] Yin Q.-Q., Jiao W.-W., Han R. (2014). Rapid diagnosis of childhood pulmonary tuberculosis by Xpert MTB/RIF assay using bronchoalveolar lavage fluid. *BioMed Research International*.

[25] Pang Y., Lu J., Wang Y., Song Y., Wang S., Zhao Y. (2013). Study of the rifampin monoresistance mechanism in mycobacterium tuberculosis. *Antimicrobial Agents and Chemotherapy*.

[26] Guo Q., Pan Y., Yang Z. (2016). Epidemiology and clinical characteristics of pediatric drug-resistant tuberculosis in chongqing, China. *PLoS ONE*.

[27] Jiao W., Liu Z., Han R. (2013). A country-wide study of spoligotype and drug characteristics of Mycobacterium tuberculosis from children in China. *PLoS ONE*.

[28] Jenkins H. E., Tolman A. W., Yuen C. M. (2014). Incidence of multidrug-resistant tuberculosis disease in children: systematic review and global estimates. *The Lancet*.

[29] Dorman S. E., Chihota V. N., Lewis J. J. (2012). Performance characteristics of the cepheid Xpert MTB/RIF test in a tuberculosis prevalence survey. *PLoS ONE*.

[30] Graham S. M., Ahmed T., Amanullah F. (2012). Evaluation of tuberculosis diagnostics in children: 1. Proposed clinical case definitions for classification of intrathoracic tuberculosis disease. Consensus from an expert panel. *The Journal of Infectious Diseases*.

