# Erratum to “The Feasibility of Xpert MTB/RIF Testing to Detect Rifampicin Resistance among Childhood Tuberculosis for Prevalence Surveys in Northern China”

**DOI:** 10.1155/2018/4360219

**Published:** 2018-09-06

**Authors:** Jie Lu, Huimin Li, Fang Dong, Jin Shi, Hui Yang, Shujing Han, Ping Chu, Yanlin Zhao, Wenqi Song, Yongli Guo, Shunying Zhao

**Affiliations:** ^1^Beijing Key Laboratory for Pediatric Diseases of Otolaryngology, Head and Neck Surgery, Beijing Pediatric Research Institute, Beijing Children's Hospital, Capital Medical University, National Center for Children's Health, Beijing, China; ^2^Key Laboratory of Major Diseases in Children, Ministry of Education, Beijing Children's Hospital, Capital Medical University, National Center for Children's Health, Beijing, China; ^3^Department 2 of Respiratory Medicine, Beijing Children's Hospital, Capital Medical University, National Center for Children's Health, Beijing, China; ^4^Department of Laboratory Medicine, Beijing Children's Hospital, Capital Medical University, National Center for Children's Health, Beijing, China; ^5^National Center for Tuberculosis Control and Prevention, Chinese Center for Disease Control and Prevention, Beijing, China

In the article titled “The Feasibility of Xpert MTB/RIF Testing to Detect Rifampicin Resistance among Childhood Tuberculosis for Prevalence Surveys in Northern China” [1], Drs. Wenqi Song and Yongli Guo should have been listed as corresponding authors, along with Dr. Shunying Zhao.
